# Biosurfactant-assisted bio-electrokinetic enhanced remediation of heavy metal-contaminated soil

**DOI:** 10.3389/fmicb.2024.1458369

**Published:** 2024-09-24

**Authors:** Jayaraman Narenkumar, Bhaskar Das, Subramani Abilaji, Kuppusamy Sathishkumar, Mohamad S. AlSalhi, Sandhanasamy Devanesan, Aruliah Rajasekar, Tabarak Malik

**Affiliations:** ^1^Department of Environmental & Water Resources Engineering, School of Civil Engineering (SCE), Vellore Institute of Technology, Vellore, India; ^2^Environmental Molecular Microbiology Research Laboratory, Department of Biotechnology, Thiruvalluvar University, Vellore, India; ^3^Center for Global Health Research, Saveetha Medical College and Hospital, Saveetha Institute of Medical and Technical Sciences, Saveetha University, Chennai, India; ^4^Department of Physics and Astronomy, College of Science, King Saud University, Riyadh, Saudi Arabia; ^5^Department of Biomedical Sciences, Institute of Health, Jimma University, Jimma, Ethiopia; ^6^Adjunct Faculty, Division of Research and Development, Lovely Professional University, Phagwara, Punjab, India

**Keywords:** *Bacilluscereus* EN6, biosurfactant, electrolyte, electrokinetic process, *Pseudomonas stutzeri* NA3

## Abstract

**Background:**

Environmental soil contamination is a serious problem for humans worldwide, as it causes many diseases.

**Methods:**

The present study focuses on utilizing biosurfactants produced by *Pseudomonas stutzeri* (*P. stutzeri*) NA3 and *Bacillus cereus* (*B. cereus*) EN6, as an electrolyte for removing chromium (Cr) from contaminated soil using the electrokinetic (EK) process.

**Results:**

As a result, biosurfactants produced by *P. stutzeri* NA3 and *B. cereus* EN6, being lipopeptides, increase heavy metal mobility in the EK process. The Cr removal efficiency of a novel electrolyte (biosurfactants) in the EK process was compared with that of NA3 and EN6 biosurfactants. The EK results revealed a maximum Cr removal of 75 and 70% by NA3 and EN6, respectively, at the end of 7 days.

**Discussion:**

The biosurfactant aids in the breaking down of the heavy metals that are present deeper into the soil matrix. From the metagenomics analysis, it was identified that biosurfactant changes the microbial community with an enhanced ability to remove heavy metals. The phytotoxicity assay confirms that NA3 biosurfactant solution showed 95% seed germination and can lower hazardous pollutants in the soil.

**Conclusion:**

The application of biosurfactants as a potent electrolyte for the remediation of hazardous pollutants is an integrated process. Overall, the results of this study suggest that biosurfactants can serve as an economic and efficient electrolyte in the EK process to remove Cr from polluted soil.

## Graphical abstract

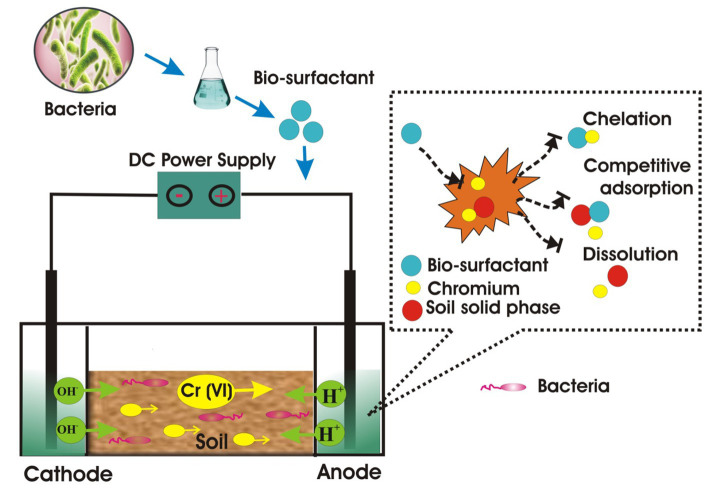

## Introduction

Environmental soil pollution has become a major issue for humans across the world. The effluents from various industries have polluted the environment with various types of harmful heavy metals (Mulligan et al., 2001; Wang et al., 2023). Among various heavy metals, Cr is widely used in numerous industries, such as Cr leather tanning, ceramics, stainless steel manufacturing, pyrotechnics, electronics, and painting and textile industries (Fonseca et al., 2012; Abilaji et al., 2023a,b). Tannery wastewater has been found to have elevated levels of chemical oxygen demand (COD), total dissolved solids (TDSs), biochemical oxygen demand (BOD), total suspended solids (TSSs), phosphate, nitrogen, and heavy metals, particularly Cr (Muthukkauppan and Parthiban, 2018; Prakash et al., 2021). However, these industries fail to implement effective Cr disposal techniques, resulting in major contamination of underground water and soil. The effects of the world’s expanding Cr pollution were known to cause neurological, renal, gastrointestinal, nasal bleeding, ulcers, skin rashes, allergies, and even human mortality (Thiele, 1995; Liao et al., 2014; Lewis et al., 2004; Mao et al., 2016).

Generally, many technologies have been proposed for the remediation of heavy metal-contaminated soils, including soil replacement, stabilization, chemical reduction, and acid washing (Devi et al., 2023). However, these methods are expensive and considered hazardous to the ecosystem. Therefore, it is necessary to develop an effective and economical technique to remediate heavy metal-contaminated soil (Taneja et al., 2023). EK remediation is an effective technique and a low-cost method for treating heavy metal-contaminated soil (Gu et al., 2018). According to Ren et al. (2014), the cost of the EK process (electrical energy) was approximately $83.3 per cubic meter of soil. Al-Hamdan and Reddy (2008) define EK remediation as the deployment of a low-intensity direct current or low potential gradient to the electrodes implanted in polluted soil. Electrolysis, electroosmosis, electromigration, and electrophoresis are the primary removal processes of EK remediation (Yeung and Gu, 2011; Zhang et al., 2016; Cameselle et al., 2021; Sathish et al., 2024). During the EK process, electrolysis produces hydrogen gas and hydroxyl ions at the cathode and hydrogen ions and oxygen at the anode. The anode-produced hydrogen ions interact with the metal cations in the soil to exchange electrons. Then, by electromigration, the desorbed metal ions move toward the cathode where the heavy metals are precipitated as oxides, hydroxides, carbonates, and other compounds by the hydroxyl ions that develop at the cathode (Santhosh et al., 2024; Priyadharsan et al., 2024). Although the heavy metals are actively precipitated causing their removal, increased accumulation of those heavy metals decreases the efficacy of cleanup. To accomplish successful remediation throughout EK procedures, an EK improvement program is frequently necessary.

In the EK approach, chelating agents and inorganic/organic acids are frequently used to remove heavy metals from soil (Santhosh et al., 2024). Apart from the aforementioned removable agents, washing chemicals, such as salts and surfactants, were also utilized to reduce surface and interfacial tension and to enhance the efficiency of heavy metal removal (Guo et al., 2016; Prakash et al., 2021). Biosurfactants exhibit higher biodegradability, less toxicity, and are more eco-friendly than chemical surfactants. Accordingly, biosurfactants are more appropriate for soil remediation. Some microorganisms (bacteria, fungi, and yeast) can produce biosurfactants as a result of metabolic activities (Kumar et al., 2021; Liepins et al., 2021; Tang et al., 2018). They also produce a number of organic acids that allow it to act as a chelating agent, increasing its potential as an electrolyte for the EK process. Hence, the present investigation is attempted to demonstrate the ability of bacterial biosurfactants (*Pseudomonas stutzeri* NA3 and *Bacillus cereus* EN6) to serve as a potential electrolyte for the EK process in removing Cr from contaminated soil.

## Methodology

### Sample collection

The heavy metal-contaminated soil sample was collected from Ranipet, Vellore, Tamil Nadu, India (latitude 12.9320°N, longitude 79.3334°E). The total Cr content of the soil was measured according to Krishna and Philip (2005) and was found to be 10.2 mg/g. This accumulation of Cr beyond the admissible limits happened during the operation of the facility, which produced sodium chromate, Cr salts, and basic chromium sulfate until 1995, and later on, the factory was closed. The samples were collected in a sterile container, transferred to the laboratory, and stored at 4°C for further studies. A physio-chemical characteristic of the soil elemental composition was analyzed using the US EPA SW 846 method 3050B. The sample was analyzed using inductively coupled plasma-mass spectrometry (ICP-MS) following acid digestion for heavy metal analysis.

### Bacterial strain and culture conditions

The bacterial strains, *P. stutzeri* NA3 (KU708859), which is a Gram-negative strain, and *B. cereus* EN6 (KR183877), which is a Gram-positive strain, were used in this study. These bacterial cultures were sub-cultured on nutrient agar (NA) and incubated for 24 h at 37°C. The colonies were plated using a streak plate technique until individual cultures were obtained, after that those colonies were inoculated in nutrient broth (pH 7.0) and then incubated for 24 h at 37°C in an orbital shaker (150 rpm) (Parthipan et al., 2017; Narenkumar et al., 2018; Tang et al., 2018).

### Production and extraction of biosurfactant

Bacterial cultures were centrifuged at 8,000 rpm at 4°C for 10 min. Then HCl was added to a supernatant to bring the pH level down to 2. The acidified supernatant was kept at 4°C overnight for precipitation. The precipitate was separated by centrifugation at 8,000 rpm for 10 min. This white precipitate produced by bacterial cultures was chosen and used to identify biosurfactants (Mahesh et al., 2006). After identification, the biosurfactant produced by the bacteria was extracted using a separating funnel. A total of 65 mL of chloroform, 15 mL of phenol, and 50 mL of bacterial culture were added to the separating funnel and kept at room temperature for 10 min. After separation, three layers were formed from which the bottom layer of biosurfactant was collected and further used for screening by different methods (the drop collapse method and the oil spreading method).

### Characterization of biosurfactant

The extracted biosurfactant was characterized using Fourier transform infrared spectroscopy (FTIR) and gas chromatographic-mass spectroscopy (GC-MS). FTIR (PerkinElmer, Nicolet Nexus-470) was used to qualitatively characterize the functional groups of the surfactant that was extracted from *P. stutzeri* NA3 and *B. cereus* EN6. The biosurfactant (10 mg) was mixed with 5% HCl-methanol reagent for GC-MS analysis. Using a Shimadzu QP2010 Ultra Rtx-5Sil MS (30 m × 0.25 mm ID × 0.25 μm) GC-MS, 1 μL of the sample was injected after the reaction was quenched with the injection of 1 mL of sterile H_2_O (Parthipan et al., 2017).

### Electrokinetic remediation

**Figure 1:**
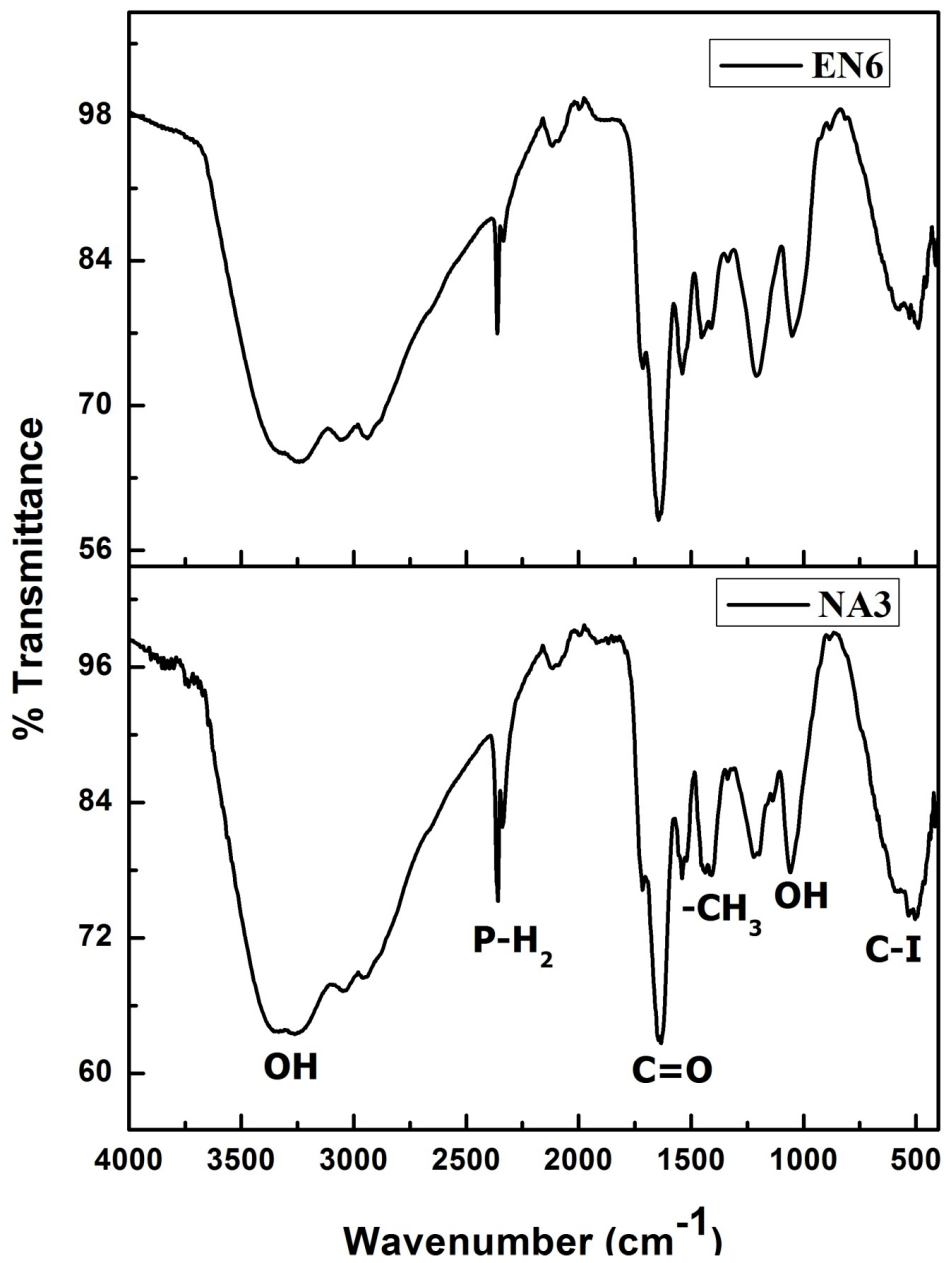
FTIR spectrum of biosurfactant isolated from *P. stutzeri* NA3 and *B. cereus* EN6.

The EK test setup and cell details are presented in Figure 1. The EK apparatus was composed of three chambers: a soil chamber measuring 30 × 5 × 5 cm (l × w × h) and two electrode chambers measuring 4 × 160 × 200 cm (l × w × h) (Sarankumar et al., 2020). To stop soil from seeping into the electrode chamber, two sheets of cellulose filter paper were placed between the three chambers. According to Marshall and Haverkamp (2012) and Prakash et al., 2021), a titanium-coated iridium oxide mesh measuring 10 cm in width and 10 cm in height was utilized as the anode, while stainless steel measuring the same was utilized as the cathode electrode (Prakash et al., 2021). This electrode was found to be corrosive-resistant and showed better electrocatalytic activity for chlorine estimation. Two electrolytes (biosurfactant solution) were used in the EK testing. A total of 600 g of dry soil was soaked in electrolyte solutions in the soil chamber for 3 days before each EK test, and the anode and cathode chambers were filled with the tested electrolytes. For 7 days, EK analysis was carried out at a constant direct current electric potential of 30 V. In order to avoid creating a hydraulic gradient in the soil column, fresh electrolyte solutions were introduced to the anode chamber every 2 days and the overflowing solution was removed from the cathode chamber during an EK procedure (Al-Hamdan and Reddy, 2008). From the anode to the cathode side, the soil chamber was divided into five slices, numbered EKS1 through EKS5. Without using a pH control, all of the trials were carried out at ambient temperature. Every day during an EK procedure, a pH electrode was inserted directly into the soil to measure the pH of the soil in each sliced segment (EKS1 to EKS5). Following the experiment, the sliced piece of soil (EKS1 to EKS5) was taken out of the EK chamber, and the soil sample was finely ground using a mortar and pestle to prepare it for X-ray diffraction (XRD) and Fourier transform infrared (FTIR) analysis (Sarankumar et al., 2020). An inductively coupled plasma mass spectrometer (ICP-MS) was used to analyze the soil sample following acid digestion. Metagenomics was used to analyze bacterial community at the end of the experiment.

### Phytotoxicity assay

The phytotoxicity analysis was conducted to determine the toxicity of the treated/untreated contaminated soil on *Vigna radiata* (SathishKumar et al., 2017). A total of 10 seeds of *Vigna radiata* were planted into the EK-treated/untreated soil. The seed germination studies were conducted at room temperature, and the length of the root and shoot from the seed was tracked throughout (Sarankumar et al., 2020).

## Result and discussion

### Biosurfactant screening

The *P. stutzeri* NA3 and *B. cereus* EN6 were found to be good producers of biosurfactants, which were confirmed by their biosurfactant production through multiple sub-culturing and screening procedures. All biosurfactant screening techniques yielded immediate positive findings for these isolates. In particular, drops collapsing within 30 s confirmed that a higher amount of the biosurfactant was present in the solution. For initial screening, the emulsification index was 80 and 78% for *P. stutzeri* NA3 and *B. cereus* EN6, respectively. Biosurfactants generated by different microorganisms are substrate-specific, emulsifying a wide range of hydrocarbons at varying speeds (Ilori et al., 2005; Parthipan et al., 2017). In oil displacement analysis, a clear zone of 2.4 cm and 2.1 cm was observed for *P. stutzeri* NA3 and *B. cereus* EN6, respectively. These findings show that the cell-free culture contains biosurfactants.

### Biosurfactant characterization

FTIR analysis was performed to determine the presence of a functional group in the biosurfactant (Figure 1). The distinctive bands at 3,309 cm^−1^ correspond to −OH bonds (Aparna et al., 2012). The peaks observed at 2,359 cm^−1^, 1,631 cm^−1^, and 1,436 cm^−1^ correspond to the P–H_2_ stretch of phosphines in phosphoserine and ester carbonyl groups (–C=O bond in COOH) (Bayoumi et al., 2010; Parthipan et al., 2017). The absorption peaks at 1,057 cm^−1^ and 534 cm^−1^ show the presence of the O–H (carboxylic acids) and C–I (carbon-iodine) bonds, respectively. Based on this observation, *P. stutzeri* NA3 and *B. cereus* EN6 were produced as biosurfactants, which is also supported by the findings of Rodrigues et al. (2006).

### GC-MS analysis

**Figure 2:**
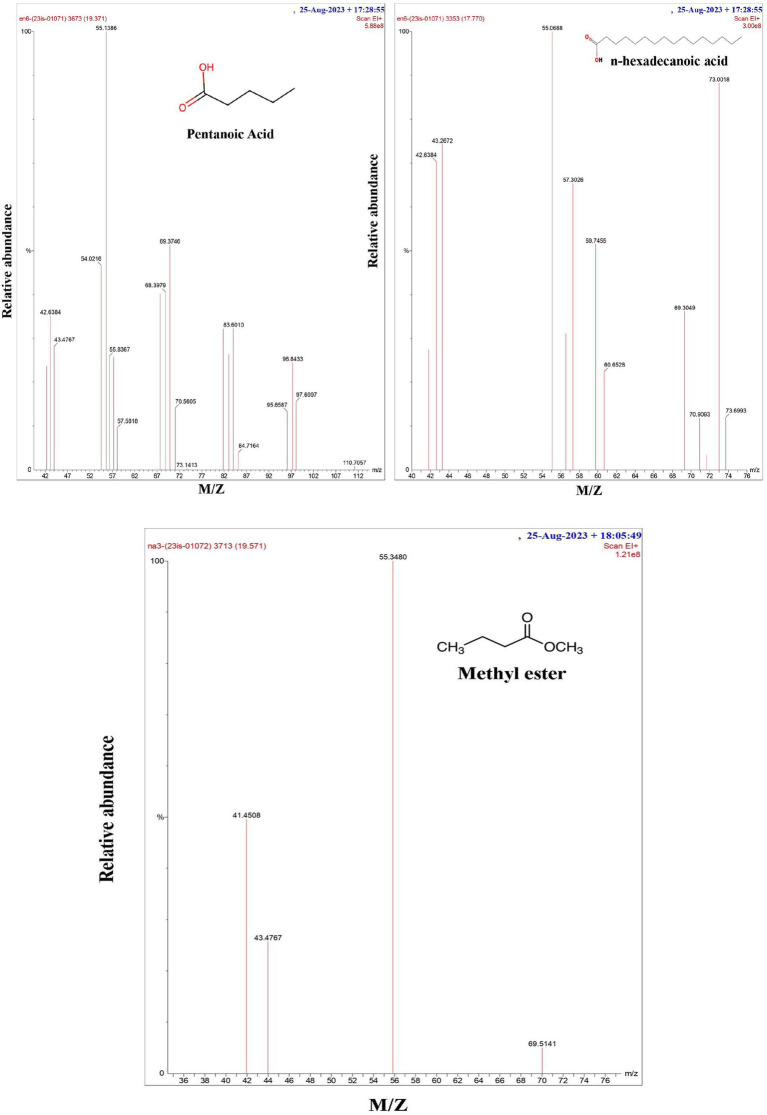
GC-MS spectrum of the biosurfactant isolated from *P. stutzeri* NA3 and *B. cereus* EN6.

The finding from the gas chromatography study revealed (Figure 2) that the biosurfactant extracted from both bacterial strains contains hexadecanoic acid and methyl ester (a fatty acid). According to Kiran et al. (2010) and Hien et al. (2013), *P. stutzeri* NA3 and *B. cereus* EN6 included fatty acids, such as hexadecanoic acid, pentanoic acid, and methyl ester with retention times (RTs) of 17.77, 19.37, and 19.50 and MWs of 256, 254, and 184, respectively. According to Deshmukh et al. (2012), Bacillus developed a biosurfactant that was essentially lipopeptide in nature. Tsui et al. (2022) state that many organic acids and metabolic products are produced by microorganisms; these chemicals stay in the solution (electrolyte) and contribute to its high conductivity. Because of this, microbes may effectively reduce the pH of the anode and create organic acids, which can combine with heavy metals to enhance their mobility.

### Electrokinetic remediation

EK experiment for Cr (VI) remediation was conducted in the customized cell setup as mentioned above. The ICP-MS technique was used to evaluate the residual amounts of total Cr by EK. The level was found to decrease from 44,615 mg/kg to 13,523 mg/kg (70%) and 11,390 mg/kg (75%) for *P. stutzeri* NA3 and *B. cereus* EN6, respectively. As previously reported, 63.34% of Cr was removed from the soil sections by using distilled water (Yan et al., 2023). The obtained results showed that 75% was reduced by electrolytes (NA3 biosurfactant) in the approach. The results revealed that Cr removal was enhanced using biosurfactant as an electrolyte, indicating that the biosurfactant binds to Cr (chelation) to form micelles, which enhances the electromigration process to remove this heavy metal from the soil. It is important to note that synthetic surfactants may have irreversible effects on soil toward the loss of essential nutrients and organic matter. However, this biosurfactant can overcome this disadvantage and it also has an added advantage of using eco-friendly, biodegradable material for the removal of heavy metals. Researchers have identified several microorganisms as potential biosurfactants that operate extremely well in removing heavy metals (Ayangbenro and Babalola, 2020; Lopes et al., 2021; Ravindran et al., 2020). Earlier studies reported that biosurfactants contain both carboxyl and hydroxyl groups, which were able to form stable complexes with heavy metal ions, complexes such as these facilitate heavy metal mobilization and migration.

**Figure 3:**
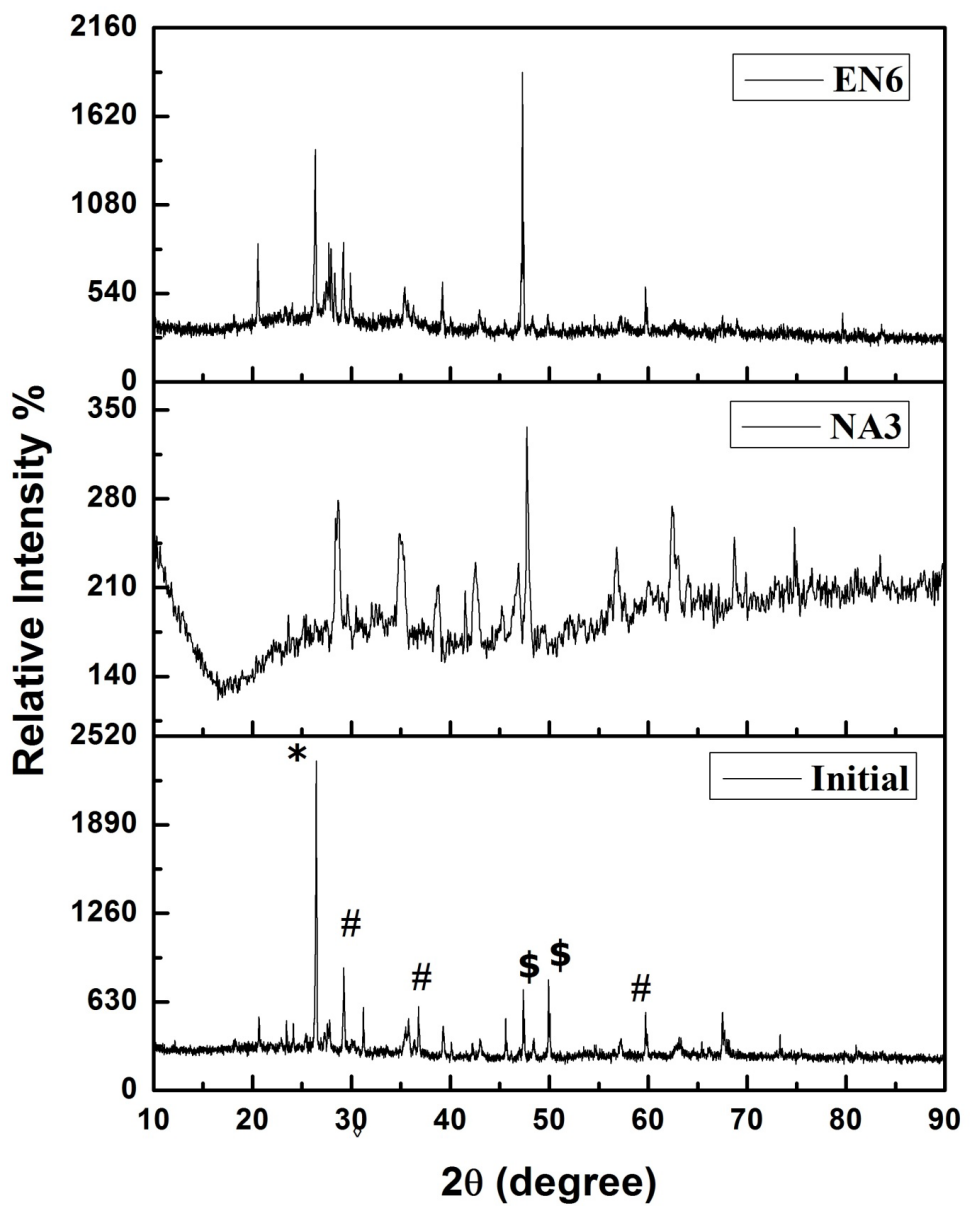
X-ray diffraction patterns of the soil before and after the EK experiment ^*^CrOCl, ^#^KCr_3_O_8_, and ^$^C_4_H_16_Cr_2_CuN4O_7_.

Figure 3 shows the XRD analysis of the untreated (control) and treated (*P. stutzeri* NA3 and *B. cereus* EN6) soil samples. Cr (VI) in the form of CrOCl, KCr_3_O_8_, and C_4_H_16_Cr_2_CuN_4_O_7_ was found in the untreated sample (initial) according to the XRD pattern. In contrast, the EK-treated soil samples (*P. stutzeri* NA3) show a decreased intensity of peaks and the presence of some other additional peaks when compared to the control and *B. cereus* EN6 due to ions that may be extracted with acid and dissolved soil components (Xue et al., 2017). From this analysis, more contaminants were found to be dissolved, and electro-kinetic remediation was found to be more suitable for acid- and water-soluble ions.

**Figure 4:**
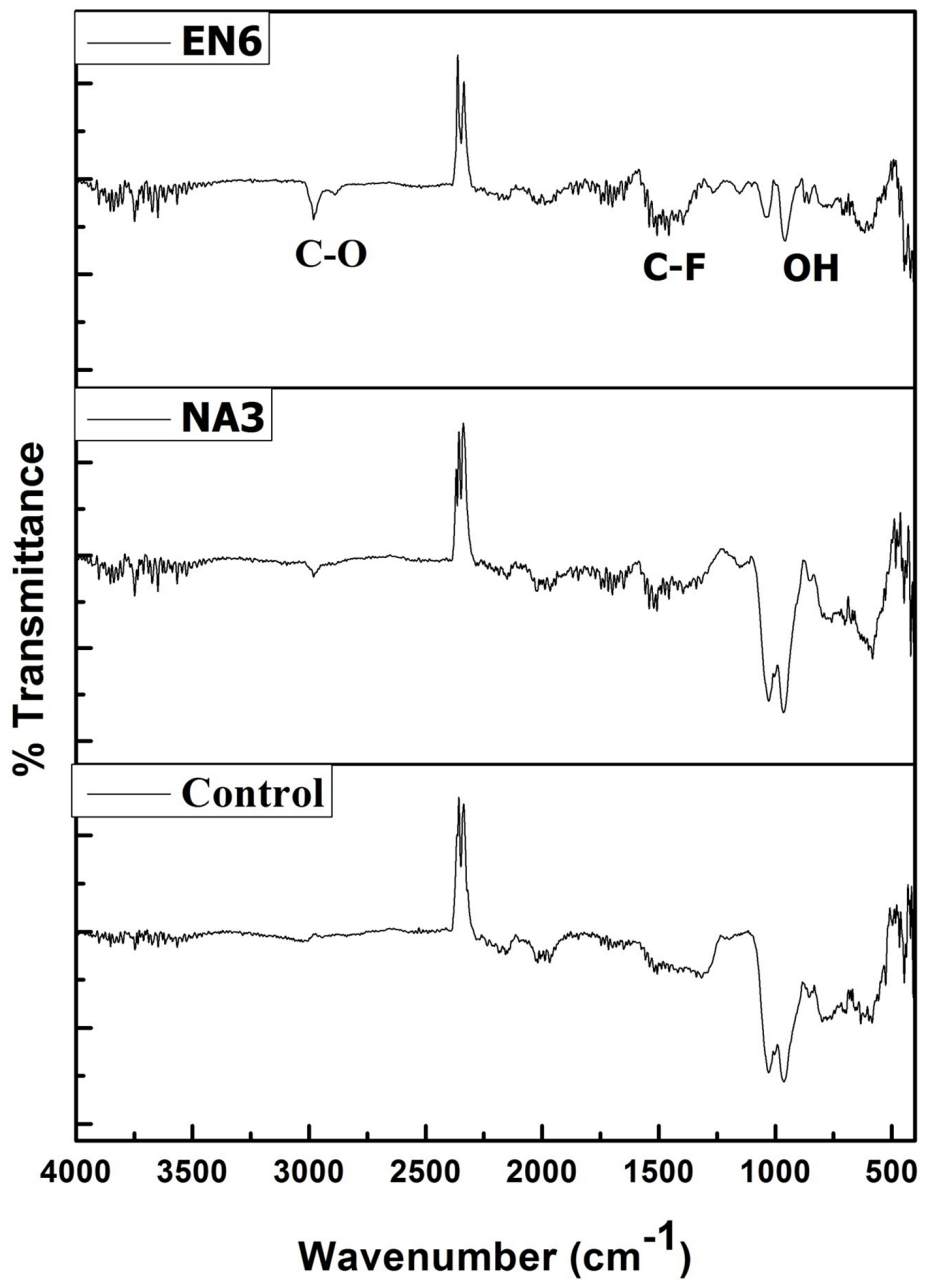
FTIR patterns of the soil before and after the EK experiment.

The FTIR spectrum of the before and after EK analysis (control, *P. stutzeri* NA3, and *B. cereus* EN6) of the soil is shown in Figure 4. In treated soil, the peak at 2,982 cm^−1^ corresponds to the presence of the carboxylic (C–O) group, which was due to alcohol groups being transformed into carboxylic groups during the reduction of Cr (VI) to Cr (III) (Bandara et al., 2020; Santhosh et al., 2024; Abilaji et al., 2023a,b). The peak at 1,986 cm^−1^ may be related to the soil’s clay mineral composition. The C–F stretch of the alkyl halide has a peak at 1,506 cm^−1^. A metal oxide hydroxide is indicated by narrow peaks at 964 and 599 cm^−1^ (Anandaraj et al., 2017). Conversely, the untreated control soil’s absorption peaks demonstrated a significant variation in peak intensities when compared to the treated soil, indicating that the Cr remediation process facilitated by the *P. stutzeri* NA3 and *B. cereus* EN6 bacterial surfactants was successful. Functional groups of lipopeptide biosurfactant bind to the Cr heavy metal ion through chelation, complexation, and electrostatic adsorption mechanisms. First, the heavy metal gets detached from the contaminated soil, which then associates with biosurfactants to form micelles (Peng et al., 2009). As a result of their low toxicity, biodegradable nature, and low environmental footprint, biosurfactants are gaining a great deal of attention worldwide.

### Bacterial diversity analysis

**Figure 5:**
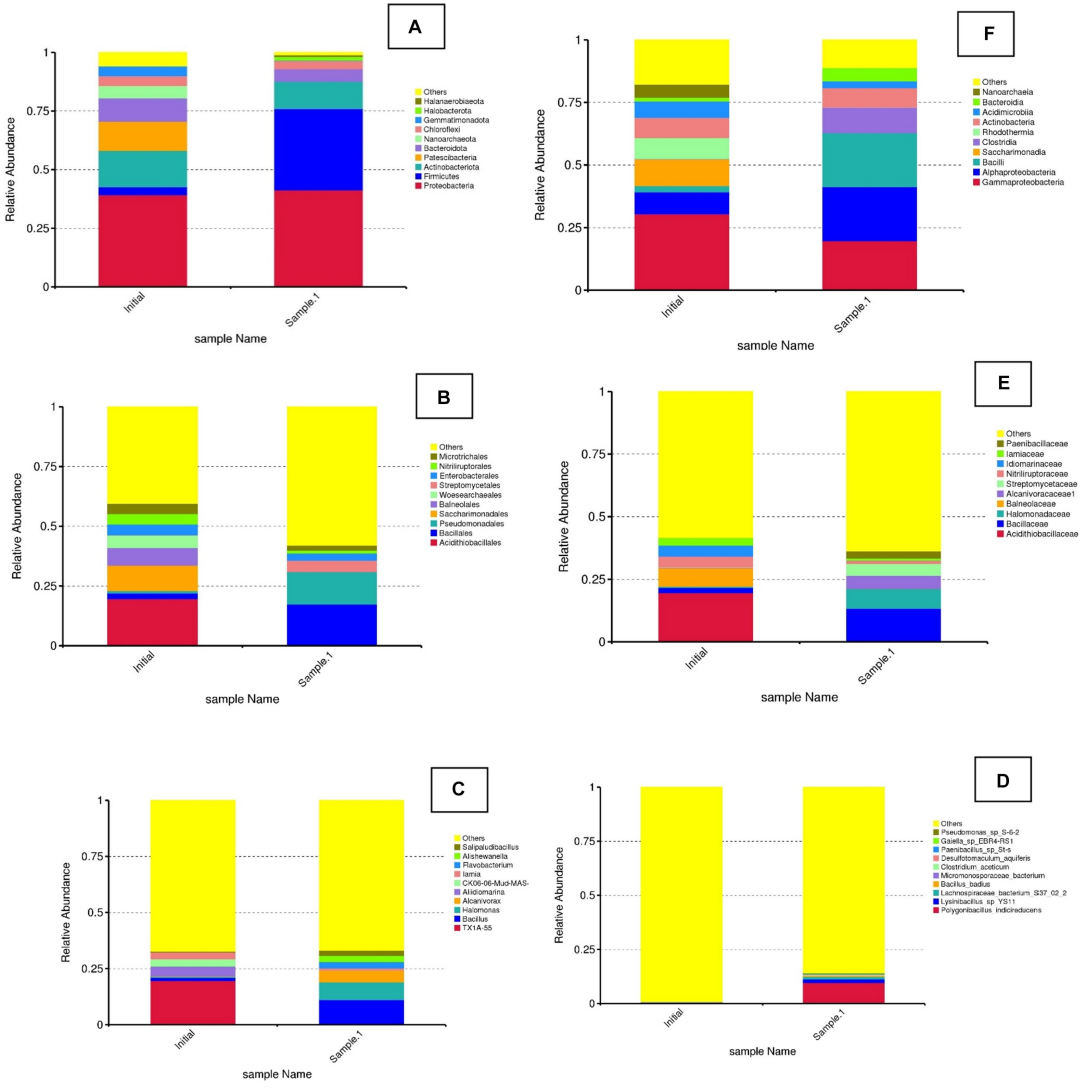
Relative abundance of bacterial diversity at phylum, class, order, family, genus, and species level in the initial and treated (EK). **(A)** Phylum. **(B)** Family. **(C)** Genus. **(D)** Species. **(E)** Order. **(F)** Class.

Figure 5 illustrates the relative abundance of different bacterial phyla in two samples, labeled as initial and sample 1 (Biosurfactant EK treatment). The plot indicates a comparison of microbial community composition before and after EK treatment. The initial sample shows the most abundant phylum as *Proteobacteria*, followed by *Actinobacteriota*, *Patescibacteria*, and others. *Firmicutes* and other phyla (*Bacteroidota*, *Nanoarchaeota*, *Chloroflexi*, etc.) were found to be present in smaller proportions. Sample 1 shows there was a noticeable shift in the microbial community composition. *Proteobacteria* and *Firmicutes* show significant changes, with *Firmicutes* becoming much more dominant in sample 1. Other phyla, such as *Actinobacteriota* and *Patescibacteria*, exhibit variations in their relative abundances. Bio-electrokinetic remediation is an emerging technology that combines bioremediation and EKs to enhance the removal of heavy metals, such as Cr, from contaminated soils. This approach leverages microbial activity and the application of electric fields to mobilize and degrade contaminants. The observed changes in microbial community structure between the initial and BEK samples were crucial for understanding the effectiveness of bio-electrokinetic remediation. Specific bacterial phyla, such as *Proteobacteria* and *Firmicutes*, were known to play vital roles in metal reduction and detoxification processes. Studies have shown that certain strains of *Proteobacteria* can reduce Cr(VI) to the less toxic Cr(III), facilitating its removal from the soil (Zhu et al., 2017). The application of an electric field can increase the mobility of Cr ions in the soil, making them more accessible to microbial degradation. This process can also enhance the transport of nutrients and electron donors to the microbial populations, boosting their activity and efficiency (Acar and Alshawabkeh, 1993, Arulpraksh et al., 2021). The integration of bioremediation with EK techniques can result in synergistic effects, leading to improved remediation outcomes compared to traditional methods.

In the class level of bacterial diversity, the initial sample was found to have the most abundant class as *Gammaproteobacteria*, followed by *Alphaproteobacteria*, *Bacilli*, and others. *Clostridia*, *Saccharimonadia*, and other classes (*Acidimicrobiia*, *Actinobacteria*, etc.) were present in smaller proportions. In sample 1, there was a noticeable shift in the microbial community composition. *Alphaproteobacteria* and *Bacilli* show significant changes, with *Bacilli* becoming more dominant in sample 1. Other classes, such as *Clostridia* and *Saccharimonadia*, exhibit variations in their relative abundances.

Whereas in the order level, in the initial sample, the most abundant order was “Others,” followed by *Acidithiobacillales*, *Bacillales*, and *Pseudomonadales*. Other orders, such as *Saccharimonadales*, *Balneolales*, and others, were present in smaller proportions. In sample 1, there was a noticeable shift in the microbial community composition. *Pseudomonadales* and *Bacillales* show significant changes, with *Pseudomonadales* becoming much more dominant in sample 1. Other orders, such as *Acidithiobacillales* and *Saccharimonadales*, exhibit variations in their relative abundances. The observed changes in microbial community structure between the initial and EK samples are crucial for understanding the effects of specific treatments or environmental changes. This is particularly relevant in the context of bioremediation, where microbial communities play a vital role in degrading and detoxifying contaminants. The shift in microbial community composition suggests that the treatment or condition applied to sample 1 has influenced the relative abundance of different bacterial orders. *Pseudomonadales*, known for their versatile metabolic capabilities and resistance to heavy metals, have become more dominant in sample 1. This indicates their potential role in bioremediation processes (Mrozik et al., 2010). The decrease in *Acidithiobacillales*, which were typically associated with acidic environments and sulfur metabolism, might indicate a change in environmental conditions or the successful removal of specific contaminants that these bacteria thrive on Johnson and Hallberg (2009). *Pseudomonadales*: Pseudomonas species were well-known for their ability to degrade a wide range of organic pollutants and heavy metals. Their increased abundance in sample 1 suggests their active role in the bioremediation process. Recent studies have highlighted their effectiveness in Cr reduction and detoxification (Raja et al., 2020). *Bacillales*: The members of this order, including *Bacillus* species, were also important in bioremediation due to their ability to produce spores, which make them resilient in harsh conditions. They can also produce enzymes that degrade pollutants (Müller et al., 2012).

At the genus level, the results showed that in the initial sample, the most abundant group is “Others,” followed by TX1A-55, *Bacillus*, and *Halomonas*. Other genera, such as *Alcanivorax*, *Aliidiomarina*, and others, were present in smaller proportions. In sample 1, there was a noticeable shift in the microbial community composition. *Bacillus* and *Halomonas* show significant changes, with *Bacillus* becoming more dominant in sample 1. Other genera, such as TX1A-55 and *Alcanivorax*, exhibited variations in their relative abundances. *Halomonas* bacteria were known for their ability to survive in high-salinity environments and their potential in bioremediation of saline and heavy metal-contaminated sites (Nieto et al., 1993).

At the species level, the most abundant group was Others, indicating a diverse set of species that were not individually listed. Specific species, such as *Pseudomonas* sp. S-6-2, *Paenibacillus* sp. and *Polygonibacillus indicireducens*, were present in smaller proportions. In sample 1, there was a noticeable shift in the microbial community composition. The diversity seems to decrease, with specific species, such as *Polygonibacillus indicireducens*, *Pseudomonas* sp. S-6-2, and *Lysinibacillus* sp. YS11, becoming more prominent. *Lysinibacillus* spp. known for their ability to produce spores and survive in harsh environments. *Lysinibacillus* species have shown potential in heavy metal bioremediation (Raja et al., 2020). They can reduce toxic metals and facilitate their removal from contaminated environments. *Paenibacillus* spp. are known for their nitrogen-fixing abilities and production of antimicrobial compounds. They also play a crucial role in the degradation of organic pollutants and bioremediation (Grady et al., 2016).

### Phytotoxicity assay

The phytotoxicity of untreated and treated plants was evaluated by *Vigna radiata*. The untreated soil showed no germination, whereas the *P. stutzeri* NA3 biosurfactant solution showed 95% germination, whereas 70% of the *B. cereus* EN6 solution did the same. This was confirmed by measuring the length of the shoot and root in the appropriate soil. It is confirmed that the treated *P. stutzeri* NA3 biosurfactant solution grows the seeds more effectively than the *B. cereus* EN6 and untreated soil because the treated soil was less hazardous. This method serves as a potential electrolyte for the EK process for the remediation of Cr from contaminated soil.

## Conclusion

Biosurfactant was determined to be a novel electrolyte for removing Cr from soil through an EK process. The study’s findings showed that at the end (7 days) of the EK process, *P. stutzeri* NA3 biosurfactant exhibited significantly higher Cr removal efficiency (75%) than *B. cereus* EN6 (70%). From the phytotoxicity assay, *P. stutzeri* NA3 biosurfactant solution showed 95% seed germination. Hence, it can be said that modifications to the microbial community structure result in an increase in the efficacy of heavy metal removal and that NA3 biosurfactant may be used as an electrolyte for EK applications. However, further investigation is required to determine whether biosurfactants can be used to remove other emerging pollutants.

## Data Availability

The original contributions presented in the study are included in the article/supplementary material, further inquiries can be directed to the corresponding authors.
